# Biosignals learning and synthesis using deep neural networks

**DOI:** 10.1186/s12938-017-0405-0

**Published:** 2017-09-25

**Authors:** David Belo, João Rodrigues, João R. Vaz, Pedro Pezarat-Correia, Hugo Gamboa

**Affiliations:** 1 LIBPhys (Laboratory for Instrumentation, Biomedical Engineering and Radiation Physics), Faculdade de Ciências e Tecnologia, Universidade Nova de Lisboa, 2829-516 Caparica, Portugal; 2 Laboratory of Motor Behaviour, CIPER, Faculdade de Motricidade Humana, Universidade de Lisboa, Estrada da Costa, 1499-002 Cruz Quebrada - Dafundo, Portugal; 3 Universidade Europeia, Laureate International Universities, Lisbon, Portugal; 4 Benfica Lab, Sport Lisboa e Benfica, Lisbon, Portugal

**Keywords:** Neural networks, DNN, GRU, Synthesis, Biosignals, ECG, EMG, RESP

## Abstract

**Background:**

Modeling physiological signals is a complex task both for understanding and synthesize biomedical signals. We propose a deep neural network model that learns and synthesizes biosignals, validated by the morphological equivalence of the original ones. This research could lead the creation of novel algorithms for signal reconstruction in heavily noisy data and source detection in biomedical engineering field.

**Method:**

The present work explores the gated recurrent units (GRU) employed in the training of respiration (RESP), electromyograms (EMG) and electrocardiograms (ECG). Each signal is pre-processed, segmented and quantized in a specific number of classes, corresponding to the amplitude of each sample and fed to the model, which is composed by an embedded matrix, three GRU blocks and a softmax function. This network is trained by adjusting its internal parameters, acquiring the representation of the abstract notion of the next value based on the previous ones. The simulated signal was generated by forecasting a random value and re-feeding itself.

**Results and conclusions:**

The resulting generated signals are similar with the morphological expression of the originals. During the learning process, after a set of iterations, the model starts to grasp the basic morphological characteristics of the signal and later their cyclic characteristics. After training, these models’ prediction are closer to the signals that trained them, specially the RESP and ECG. This synthesis mechanism has shown relevant results that inspire the use to characterize signals from other physiological sources.

## Background

Biosignal synthesis has been applied in biomedical engineering research to mimic the chemical and physical processes that can be measured with sensors and characterized by their quantification. Each type of biosignal has a characteristic morphology depending on the measured surface or organ, the source, i.e. the individual that generated it, the contamination noise and, in some cases, the pathology.

This paper proposes the application of a deep neural networks (DNN) to accurately synthesize the morphologies of a biosignal. The hypothesis is that if the created models are capable of generating clean signals, apart from replacing the unrecognizable signals due to contamination of noise, but they could also evaluate the distinction between types of signals and, if the signal topology permits, its source. This capacity will be able to unlock novel algorithms, not only for signal denoising and reconstruction, but also for event detection, classification and validation. The DNN architecture is a fundamental key in this study, since it can learn from the morphology itself, not requiring the input of more features nor the compatibility for one specific signal, unlike other methods existent in the bibliography.

The remainder of this paper will follow the explanation of the morphology of the three biosignals that were used to validate the proposed architecture, followed by the review of related works and the structure of the gated recurrent units (GRU), the main component of the DNN architecture used and a specific application of these, the character language model, which is an inspiration for the creation of the proposed architecture. “Dataset” and “Methods” sections will cover the dataset and used methods, “Results” and “Discussion” sections will provide the experimental results along with their detailed discussion. Conclusion and future work will be presented in “Conclusion and future remarks” section.

### Signal morphology

**Fig. 1:**
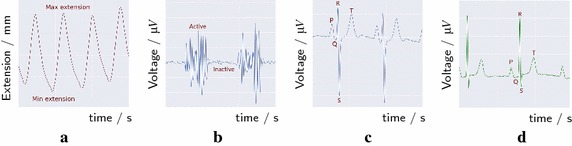
Four different acquired biosignals: **a** is an example of a respiration signal (RESP) signal; **b** is an example of an electromyogram (EMG) signal; and **c**, **d** are two electrocardiogram (ECG) signals from two different subjects

The Greek etymology term morphology is: *morph*—‘shape’, ‘form’ and *logy*—‘study of’, therefore it is the study of shapes or forms. In this paper, the definition used for biosignal morphology is the shape of it’s graphical representation, visualized and perceived by the human eye in terms of periodicity, amplitude, structure, disruptions and clearness in the form of the signal.

For example, the RESP signal presented in Fig. 1a was recorded in the thorax region by a pneumatic respiration transducer, i.e. an extensiometer embedded in a elastic belt that captures the changes in volume. The extension and compression of the chest events while breathing are transcribed in the small changes in its frequency when breathing normally.

An EMG wave is a signal with a high frequency with periodic change of amplitudes (Fig. 1b), each burst is correlated with the muscle activation from the neurophysiological events that precede the muscle contraction.

The family of biosignals presented in Fig. 1c, d, denominated ECG. The characteristic form of these biosignals may be described as being a baseline that oscillate in a cyclic pattern of five different waves, reflecting each phase of the heart beat: P—corresponding to the atrial contraction; the QRS complex— responsible for the contraction of the ventricles; and, T—consequence of the ventricular relaxation [1–3].

The morphology disparity between biosignals of the same family may reside on individual traits, different electrode placement in relation to the measured organ, artifacts (caused by internal or external sources), noise or pathological events. Due to the increase of external devices that measure biosignals, the level of noise corrupting the signals is substantial making them unreadable.

### Related work

The applicability of synthesized signals range from denoising, reconstruction of unreadable signal to event detection, classification and validation and the most relevant research is on the generation of EMG and ECG.

The existent approaches in EMG reproduction include the use of a sum of diphasic waves [4, 5], the implementation of a random EMG tonic wave and multiplication by a sinus wave [6] or using autoregressive models and mixing them with gaussian noise [7].

In the ECG end, various research articles rely on its theoretical expression, such as the combination of cosine waves[8], the coupling of differential equation [1] or using delayed harmonic waves [9]. After the parametrization of a model, adopting signal processing and machine learning methods, one can synthesize signals by exploiting its prediction power. For instance, features may be extracted with wavelet transform [10, 11] and Hilbert-Transform [12, 13] and the ECG may be generated using dynamic time warping [10], hidden Markov models (HMM) [11], polynomal approximation [12] or artificial neural networks (ANN) [13]. Atoui et al. [14] uses a multilayer ANN but feeding it with raw signal extracted from a 12-Led ECG considering five derivations and establishing a relationship between them.

The standard for cardiac monitors defined an artificial wave based on characteristic parameters, such as the QRS amplitude and time, as a standard for designing and validating event detectors [15].

### Deep neural networks and gated recurrent units

The ANN algorithms learn from data by optimizing multiple parameters, which turns them more capable of solving specific problems [16, 17]. The DNN represent the evolution of the “shallow” networks with the increase of hidden layers, complexity, computational power and learning capabilities. The long short-term memory (LSTM) was proposed by Hochreiter and Schmidhuber [18–21] as a solution to the vanishing and exploding gradient issues. This architecture has multiple gate layers concerned with memory management that are capable of learning long-term dependencies by forgetting and updating the layers state.

The GRU architecture is a simplification of the LSTM algorithm. They are both recurrent neural networks (RNN) as they may be represented as a conventional feedforward neural network, in which the next phase depends on the previous ones. In the unfolded version of the feedback loop, the sequential data is passed through each network, changing its internal state, recording the dynamic changes of the input [21, 22].

**Fig. 2:**
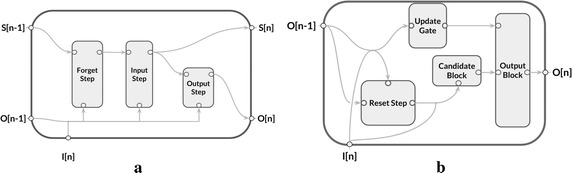
Single unit representation of the LSTM (**a**) and a GRU (**b**) neural networks (NN)

The LSTM has three gate system (Fig. 2a) that protect and control the cell state, providing the LSTM the ability to continuously write, read and reset information of the cell state [23] while the GRU architecture relies it’s efficient memory management on two gates: reset and update (Fig. 2b) [24, 25].

GRU is known for converging faster, without the cost of accuracy, in comparison to LSTM. The other advantage of this algorithm is the high prediction rates while estimating the sequence of time-series data, in several fields, without the input of an extensive amount of features, nor their selection. The promising results may be seen in several areas, such as speech [17, 26, 27], music [28, 29], audio analysis [30] and handwriting recognition [31, 32]. These architectures are also used in the area of language comprehension for text translation [33], text generation [34] and image and video description [35–37].

More detailed information is available in the “Methods” section.

### Character-level language model

In the context of learning of natural language, the GRU model has been used in the prediction of the next character in a text. From a sequence of characters in a sentence, the RNN model is capable of learning the correct structure of phrases. Graves [38] describes the example of “wikipedia experiment” where the network was able to generate text as a wikipedia template. Even though the phrases were well structured and grammatically correct, the overall article does not have any meaningful content. Just as Koski [39] relates the ECG with the syntactic expression, where words and grammar represent the patterns dictated by a set of rules, this paper explores the extrapolation of this concept into physiological signals area.

## Dataset

In order to reconstruct data from unrecognizable signals, due to noise, and for the detection of abnormal events, the model needs to learn the clean version of the signal. Accordingly the chosen dataset was based on three principles: free from noise; acquired from individuals without pathologies; and, the signal morphology must be directly interpreted by a human without any special expertise. This document uses RESP, EMG and ECG signals [40, 41].

The ECG and RESP were downloaded from the Physiobank database, which was created under the auspices of the national center for research resources of the national institutes of health [40]. The dataset contains acquisitions from the first ten people with ages between 21 and 34 years old and first ten with ages between 68 and 81 while exposed to 120 min of continuous supine resting electrocardiograph recording while watching the Disney’s movie Fantasia [41].

**Fig. 3:**
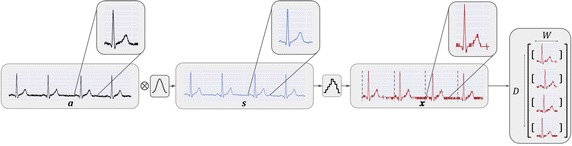
Signal pre−processing algorithm. *a* represents the raw signal, *s* the smoothed version of the signal, *x* the quantized signal, *D* the dataset dimension and *W* the size of each time window (TW)

The EMG dataset was acquired during an experience in the faculty of human kinetics (FMH), Lisbon, with the purpose of measuring muscular fatigue during cycling exercises. In this study, fourteen healthy male that were physically active, non-expert that only cycled for leisure. During the experiment the participants performed consisted in a constant power exercise maintained until task failure on a cycloergometer (Ergomedic 839E, Monark, Stockholm) while recording the EMG of the lower limbs (Fig. 3).

## Methods

The proposed DNN^1^ sequential architecture is depicted in Fig. 5. In sum, after reducing noise, quantizing and segmenting the signal, each scalar sample—$x_n$—is fed to the network. This value corresponds to the index of the embedded matrix—*E*—transforming into the column vector $\hat{x}_n$, the inner representation of the sample. The result of the three GRU layers is vector $\hat{o}_n$ that will be the input of a regression node and of a softmax function giving a probability density vector *o*. After the models are trained, resorting to RMSprop algorithm, with different signals, these are synthesized by exploiting their prediction power.

The detailed explanation of the pre-processing, model, training method, signal generation and model evaluation will be addressed in this section.

### Pre-processing

**Fig. 4:**
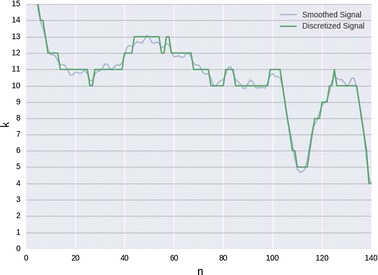
Example of a signal transformed by a quantization process. The blue and green line represent the pre-processed signal before and after quantization, respectively. *k* represents the step in which that amplitude belongs to and *n* the respective sample

The noise and dimensionality reduction are required so that the dimensions match the matrices of the network, making the pre-processing an essential step. The first stage consists on a moving average filter, that removes the lower frequencies and casual disruptions, and the convolution with a Hanning window, that removes the higher frequencies, resulting in a smoothed version of the signal. The complexity of the signal was reduced using a quantization method, where each step of the signal is represented by an integer value:

1 $$\begin{aligned} x_n = {{\mathrm{round}}}\Bigg ( \left[ \frac{ s_n-\text{min}(s) }{\text{max}(s) } \right] \cdot [S_D-1] \Bigg ) \end{aligned}$$

where the ${{\mathrm{round}}}$ function rounds the result to to the nearest integer. Consequently, *x* will be a vector where each position corresponds to its associated step $k \in \{0, 1,\ldots S_D-1\}$. Fig. 4 depicts an example of a signal’s TW before and after this process for $S_D = 16$.

The label signal *y* is simply a de-phase of the input *x* by one sample, so that ${y_n = x_{n+1}}$, where ${y_N=0}.$ For the sake of computational power, the last step of the pre-processing is the segmentation of the dataset *D* into TWs with dimension *W* and overlap of 2/3, resulting in two matrices *X* and *Y* with the same dimensions of *D* × *W*.

### Signal sample embedding

After pre-processing the signal, the input is transformed using an embedding matrix—*E*—before entering the GRU layers, common in the bibliography [43–45]. Instead of a one-hot vector to represent the input signal sample, a low-dimensional vector is used. The square matrix *E* of size ($S_D$ × $S_D$) contains all the representation vectors for each possible signal sample value $\hat{x}_{n} = E_{[:x_n]}$ considering that $x_n$ is an integer scalar, $E_{[:x_n]}$ represents the $x_n{\text{-th}}$ column vector of *E*. This matrix is used as a dictionary that gives an image vector $\hat{x}_{n}$ of the scalar $x_n$. The $\hat{x}_{n}$ is the input vector of size $S_D$ of the first GRU node as the $n{\text{-th}}$ sample of the TW. The matrix *E* is a learning parameter that starts with initial random values but will adjust while training the model [43, 44].

### Gated recurrent unit layers

The architecture is composed by a sequence of three GRU layers. As stated by Cho et al. [46, 47], each GRU layer—$GRU_l$—comprises three gates, each one responsible for resetting ($r{\text{-gate}}$) or updating ($z{\text{-gate}}$) the current state.

**Fig. 5:**
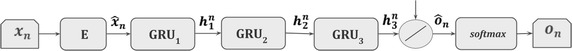
Sequential DNN model. This model comprises one embedded layer—*E*, three GRU layers, one regression node and one *softmax* function. The variables above the arrows represent the output and the input of the previous and the following node, respectively

First, the reset gate—*r*—has the following general equation

2 $$\begin{aligned} r = \sigma \left( \left[ W_r x_n \right] + \left[ U_r h_{n-1} \right] \right) \end{aligned}$$

where $\sigma$ is the logistic sigmoid function, ${x}_n$ is the input vector (Fig. 5), $h_{n-1}$ is the previous GRU memory state and the weight matrices $W_r$ and $U_r$ of size ($H_D$ × $H_D$) are learned through training. Similarly, the update gate *z* is computed by

3 $$\begin{aligned} z = \sigma \left( \left[ W_z {x}_n \right] + \left[ U_z h_{n-1} \right] \right) \end{aligned}$$

The output step is bounded with the state of the network, which is defined by the outcome of the update gate if the hidden cell is updated or not. The activation of the unit $h_n$ is computed by

4 $$\begin{aligned} h_n = \left[z_n \cdot h_{n-1} + {\left(1-z_n\right) }\widetilde{h}_{n}\right] \end{aligned}$$

where

5 $$\begin{aligned} \widetilde{h} = \phi \left( \left[ W_h {x}_n \right] + \left[ U_h (r_n \cdot h_{n-1}) \right] \right) \end{aligned}$$

$\phi$ represents the hyperbolic tangent function. When *r* is close to zero, *h* is computed ignoring *h*, using only ${x}_n$ value. The candidate for the next state results from the compilation between the new inputs and previous cell states. In the reset step, the candidate is allowed to forget the cell’s previous states, leaving the new inputs as the main guidelines for posterior outcomes. Therefore, *r* is the gate responsible for effectively replace irrelevant state information. Each hidden unit has their own *r* and *z* gates, and, consequently, will learn to capture the biological signal’s time-dependent features.

The units that have a reset gate more frequently activated capture short-term dependencies, while the units that often activate the update gate are correlated with long-term dependencies [46, 47]. After $GRU_3$, the model’s output will be computed by a regression node computed as follows:

6 $$\begin{aligned} \hat{o}_{n,\,j} = b_{o,\,j} + \sum _{n=1}^W V_{j,\,h}\,h_{j,\,n}^{(3)} \end{aligned}$$

where $V_{h^j}$ is the weight matrix, of size ($H_D$ × $S_D$), connecting the $j{\text{-th}}$ hidden layer with the output of the layers and the output of the DNN model.

The vector $\hat{o}$ is then normalized with a *softmax* function. The output vector *o* is the probability density function of the next sample—$x_{n+1}$—of having the value-*k*:

7 $$\begin{aligned} o_n^k = Pr(y_{n} = k|x_n) = \frac{\exp (\hat{o}_n^k)}{\sum _{k} \exp (\hat{o}_n^k)} \end{aligned}$$

Since the signal is represented as a step system, where each step is represented as *k*, the output of the model is a vector with $S_D$ elements.

### Training

This typologies must follow the training procedure, where the initial parameters have a random or semi-random initialization, where the parameters are learned while reducing the error while predicting the output in relation to a fed input. The loss function quantifies the amount of errors of this prediction comparing to the corresponding labels. It was defined as the loss function to be the cross entropy loss, given by:

8 $$\begin{aligned} L(p_n, o_n) = - \frac{1}{N} \sum _{n}^{N} \left [ p_n \log o_n + \left(1-p_n\log \left(1-o_n\right)\right) \right ] \end{aligned}$$

where $p_n$ is the true density probability function for sample *n*, in this case, since $y_n$ is an integer value, $p_n$ is an “one-hot vector”, where the position $y_n$ has the value of maximum probability, against the zero in the rest of the positions. When training, the desired optimum parameter value $\hat{\theta }$ is the minimum loss, depending on the parameter values:

9 $$\begin{aligned} \hat{\theta } = {{\mathrm{arg\,min}}}_{\theta }(g) \end{aligned}$$

where the gradient *g* follows:

10 $$\begin{aligned} g = \frac{\partial L(\theta )}{\partial \theta _i} \end{aligned}$$

The minimum value of the loss function is calculated by descending in the gradient values and through Backpropagation Through Time (BPTT) [48, 49]. The BPTT algorithm is commonly used for training DNN.

The chosen method to find the gradient minimum was RMSProp, proposed by Tieleman [50] which performs a parameter update for each training example $x_n$ and label $y_n.$ The loss function is represented as $L(\theta )$ and the respective gradient as $g_t$, with respect to the parameters $\theta$. The update of the values is made iteratively and is calculated for each epoch *t* with the following equation:

11 $$\begin{aligned} \theta _{t+1} = \theta _{t} - \frac{\eta }{\root \of {E(g^2)_t + \epsilon }} g_t \end{aligned}$$

where $g_{t}$ is the gradient at epoch *t* for the parameters $\theta$, $\eta$ is the learning rate and $\epsilon$ is a smoothing term (normally of value $1 \times 10^{-8}$) that prevents the division by zero. The term $E[]_t$ is the average at epoch *t* and only depends on the previous average and the decay factor $\gamma$:

12 $$\begin{aligned} E(g^2)_t = \gamma \cdot E(g^2)_{t-1} + (1-\gamma ) \cdot g_t^2 \end{aligned}$$

where $\gamma$ is usually between 0.9 ad 0.95.

While training the dataset, each signal was divided in a fixed number of TWs batches—$B_D$.

### Signal synthesizer

After training the model, the synthesis of the signal was performed by the re-feeding the input of the model with the last prediction. Since the output is an array with the probabilities of the next sample step, the selected value is based on a probability density function, hence the predicted value is a semi-random choice.

The generated signals were based on a model that was trained with the referred three distinct signal types for each individual, totaling 54 different models.

### Model evaluation

For the evaluation of the models and to guarantee that the signals were independently modeled, the mean squared error was calculated for each batch of data for each signal and model:

13 $$\begin{aligned} \text{Error}\,(B) = \frac{1}{N\cdot B_D}\sum _{b=0}^{B-1}\sum _{n=0}^{N-1}(\hat{y_n^b}-y_n^b)^2 \end{aligned}$$

The mean square error is closer to the morphology evaluation than the cross-entropy error. The prediction error of the model that was trained by a signal should be lower in comparison to the other models.

Each signal was pre-processed and separated into a training and a testing set: 128 random TW of the first 33% of the signal were used for train; 66% of the signals were used for test. The test windows had a size of 512 samples and the number of windows dependent on the size of the signal .

The mean and standard deviation were calculated for all windows, for each signal and mo

## Results

The created DNN model was tested in three types of biological signals, as stated before: RESP (6); EMG (7); and, ECG (Figs. 8 and 9). This section also covers the information related to the learning stages of the algorithm adopting the ECG as input, since it has a characteristic morphology and easily recognizable. The prediction error for each type of signal was averaged and the error for the model of the ECG for each individual was also calculated.

**Fig. 6:**
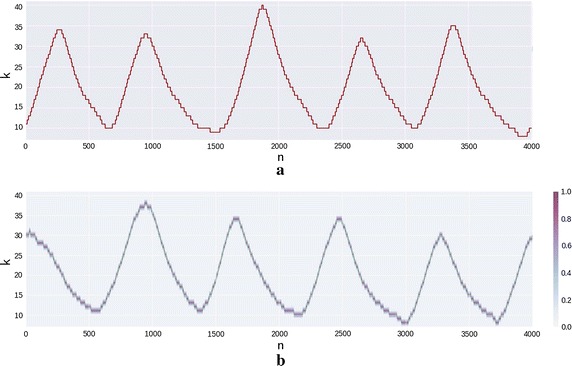
RESP prediction for subject three of fantasia dataset. **a** Depicts one TW of the pre-processed signal of the original dataset. **b** The green signal is the generated signal with the DNN model for this subject in purple the probability of each step—*k*—given by the output of the previous sample

### Resp generator

The RESP generator synthesized signal is depicted in Fig. 6. The red graphic is a segment of the pre-processed RESP that belongs to the subject three of the *Fantasia* dataset [41] and the green graphic is the synthesized version. The purple area, corresponds the the probability of each sample, and the almost invisibility is due to the high confidence of the network prediction. The used parameters were $W = 1024$, $H_D = 512$ and the $S_D = 64$. The lower frequency of this signal required a higher training window and its simplicity required less epochs for the learning process than the other two. After some try-and-error it was understood that the *W* parameter is important, because the model must encode in its states at least one full cycle of the signal in each *TW* while training.

**Fig. 7:**
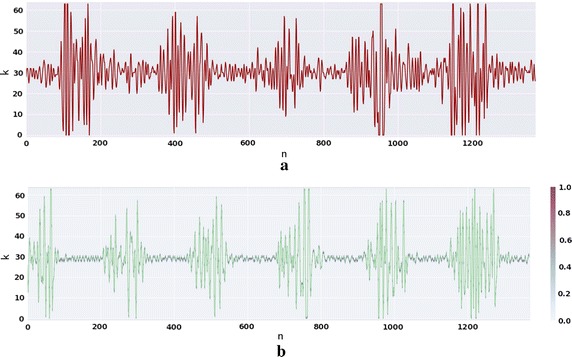
EMG prediction for subject one of the FMH dataset. **a** Depicts an example of the pre-processed signal **b** Again, the generated signal with the DNN model but with the probability in purple for each step—*k*—given by the output of the previous sample

### Electromyogram generator

Figure 7a represents an EMG signal from the *gastrocnemius medialis* muscle while pedaling in a cycloergometer, in which the active phase represents the muscle activation while pushing the pedal.

The EMG were downsampled to 250 from 1000 Hz to maintain conformity with the other models. The selected dimensions where $TW = 512$, $H_D = 512$ and the $S_D = 64$. The $H_D$ had to be increased because the wide range in frequencies needed a recipient capable of coding this information inside the network.

### Electrocardiogram generator

**Fig. 8:**
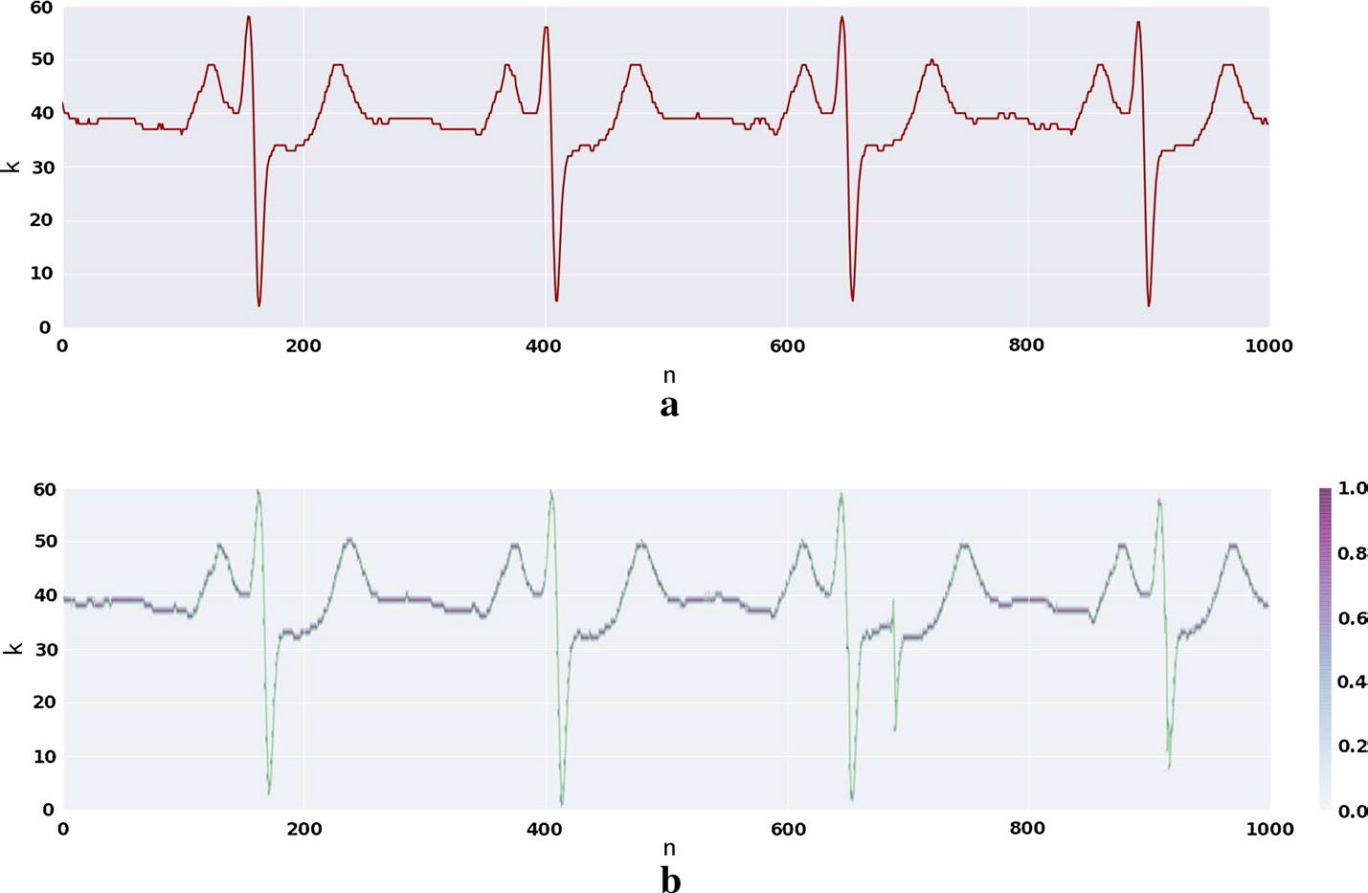
ECG prediction for subject three of fantasia dataset. **a** Depicts one TW of the pre-processed signal of the original dataset. **b** The green signal is the generated signal with the DNN model for this subject and in purple the probability of each step—*k*—given by the output of the previous sample

Training of the ECG with a high $S_D$, in the first experiences, was very hard to compute, therefore it was established that in the first stages, the algorithm would learn with lower resolutions. One representative example of the last experiments is depicted in Fig. 8 which was trained with a $W = 512$, $S_D = 64$ and $H_D = 256$. The *W* value must comprise at least one full cycle of the biosignal, since the sample frequency is 250 Hz and the period of a normal ECG is 60 beats/min.

### Model evolution

**Fig. 9:**
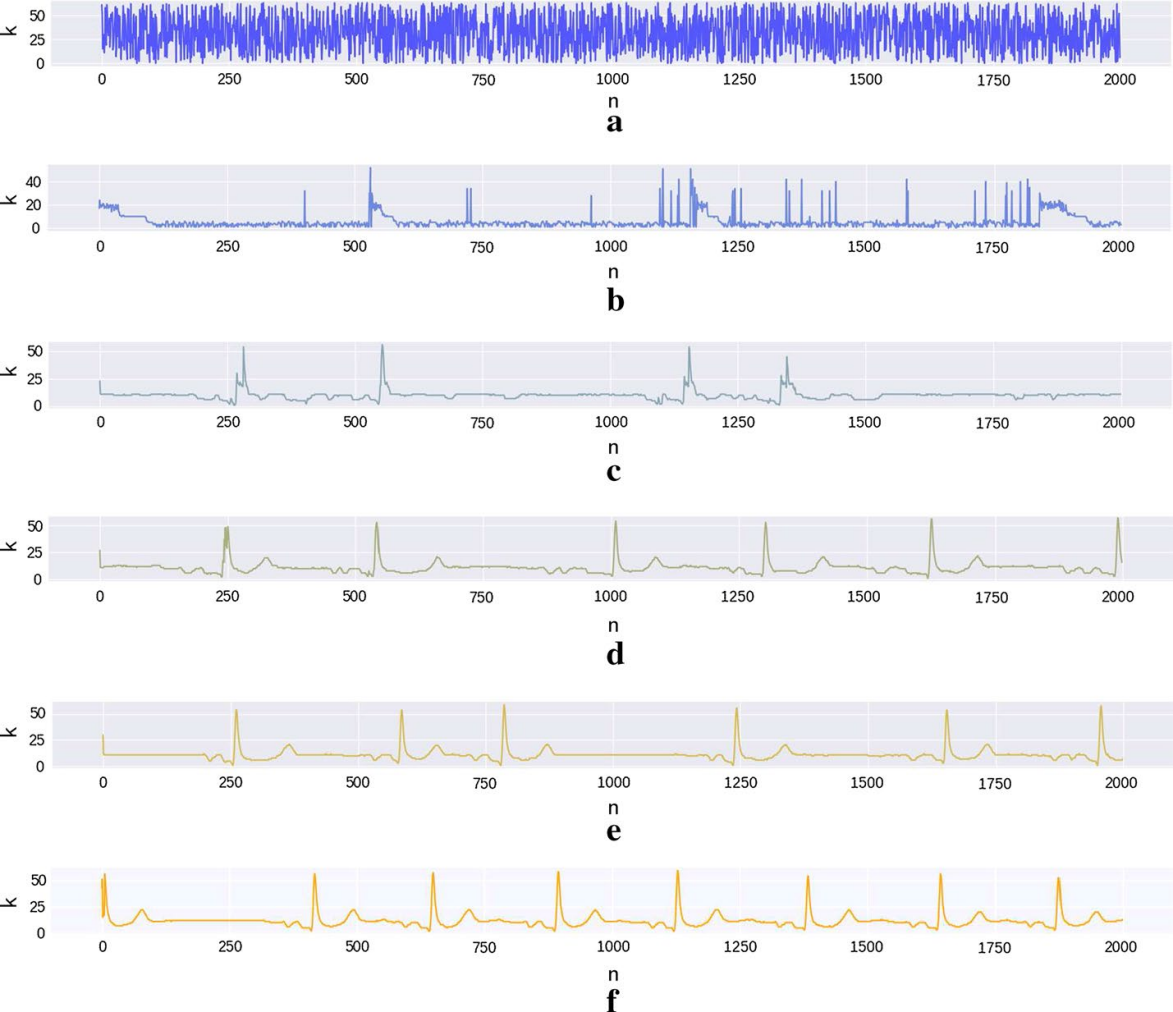
History of the ECG DNN model with $S_D = 64$ and $H_D=256$ for subject seven of fantasia dataset. Each graphic depicts the predicted signal for a model that was trained after a specific number of epochs during a specific batch of data: **a** 0 epochs; **b** 20 epochs; **c** 30 epochs; **d** 50 epochs; **e** 80 epochs; and, **f** 410 epochs

The model exemplified in Fig. 9 is the synthesis of the ECG of the individual number 7 of the *Fatasia* dataset with the parameters $W = 512$, $S_D = 64$ and $H_D = 256$. While the model was being trained, several copies were made with the purpose of having a graphical representation of how the model was learning. Therefore it is depicted six generated signals for a different number of trained epochs. For example, the first graphic (Fig. 9a) is a result of the saved model right after initialization. As for the second graphic (Fig. 9b), it is the result of the prediction of the model trained with the same batch but after 20 epochs.

### Model evaluation

**Fig. 10:**
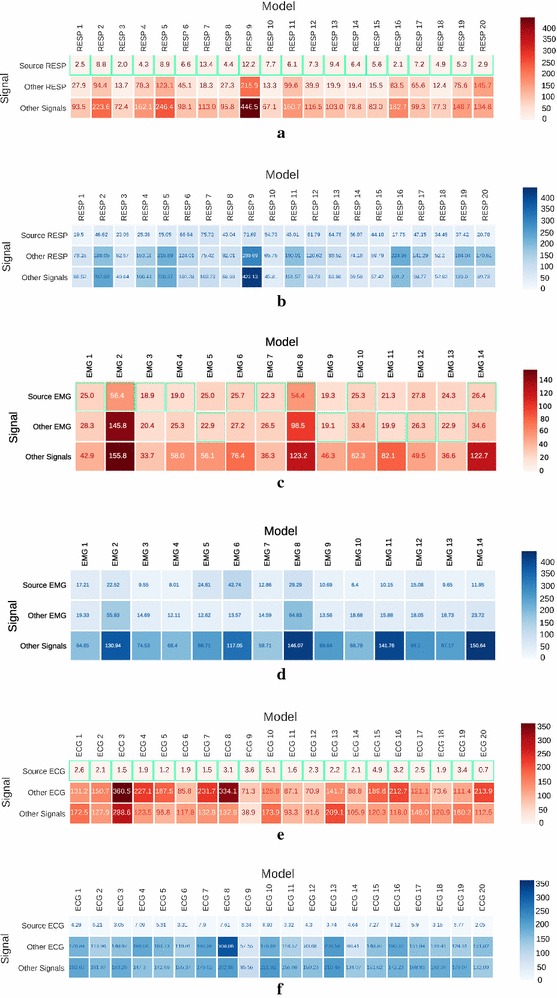
Average (**a, c, e**) and standard deviation (**b, d, f**) of the mean squared error of each model while predicting the input signal. The green square represents the lowest error for the correspondent model: **a** and **b** - RESP; **c** and **d** - EMG; **e** and **f** - ECG

For the model evaluation the prediction error for each type of signal is presented in Fig. 10. The average and the standard deviation values were calculated with the same window size ($W=512$) for each signal and model. None of the widows of the testing group were fed to any model while training. The number of TW depended on the signal size, for RESP and ECG the $B_D=3584$ for each and for EMG was $B_D=612$. The green squares represent the lowest averages for each signal each column. The first row source signal is relative to the signal that trained the correspondent model, as for the second row refers to all the other signals of the same type while the third is linked to rest of the signals.

## Discussion

While observing the RESP synthesis, depicted in Fig. 6 the model learned the patterns, the amplitude and even the small differences in frequency throughout time. The average error (Fig. 10a) is lower for the source RESP, reflecting the capacity of this algorithm to reproduce the signal that trained it. The differences from other RESP and other type of signals are also visible, even though the last are more pronounced, in parallel to the standard deviation.

In the predicted EMG (Fig. 7b), the cycles are visible and the frequency of the bursts are presented, conjugating the higher maxima after the local minima. In the synthetic version we can verify that this state machine consistently identify the activation time location. On the other hand, the bursts’ shape shows some inaccuracies, particularly in the last burst where the activation duration is clearly longer in the synthesized signal.

While analysing the error (Fig. 10c) one may realize that the EMG signals are quite similar between the source and other EMG. The reason behind this suggests various hypothesis for this fact: one is that the EMG between subjects performing the same task are quite similar; other could reside in the fact that the network did was not able to distinguish the various different frequencies in each individual, because of the inherent complexity of the signal; or, the training period ended before reaching the global minimum of the loss function. When comparing the standard deviation between the EMG data and the other type of signals, it is possible to conclude that the models are capable of synthesizing EMG that are significantly different, even if the correspondent mean, in some cases, is close.

In relation to the ECG generator, all the ECG characteristics are visible both in the original and synthesized signals. The model did not only learn the frequency and principal characteristics of the compound wave, but also the baseline at $k \simeq 40$ and the values of the local minima and maxima. The R peaks have small fluctuations in value, reflecting the original ones. It is possible to observe that after the 600th sample of the synthesized signal, that the model made an error in prediction, but it was capable of readjusting the earlier form regaining the proper morphology.

One further aspect of this ECG modulation is the fact that the network also learns the individual traits of the person. In Fig. 9 the synthesized ECG produced by the model trained with subject seven of the *Fantasia* dataset is clearly different of the one created from the subject three, depicted in Fig. 8.

The learning process of the ECG model depicted in Fig. 9, in the first epoch the model parameters were initiated with random values resulting in a sequence with a mean value and a standard deviation. After 20 epochs, the model starts to learn a few characteristics of the signal such as R spikes. Although there is no notion of frequency, there is the sense that the signal must return to a base value, in this case, of approximately $k=10$. After 30 epochs the some R peaks become more defined and some rudimentary forms of the T or P waves appear.

After 50 epochs (Fig. 9d), the model insists on introducing at least one slow wave before and after the QRS complex, and, even though it forgets at times a P or a T between two R, it doesn’t repeat these waves. Some of the R peaks do not have the final form Finally, after 80 epochs, even though there is a latency in between some waves, it is possible to see the notion of periodicity, even though it is not yet correct. The definition of the P, Q, R, S and T waves and their sequence also reflects the original ECG characteristics (Fig. 8a). And, finally, after 410 epochs, when the model was finished learning, the model can now reproduce the signal with minor differences, with the notion of frequency.

After the models evaluation, the results in Fig. 10 show that the network as higher error for the types of signal that did not train suggesting that the models recognized the type of signal that generated them. As for Fig. 10e the models were even able to reproduce with low error, each of the sources that trained them. While observing the matrix, one can speculate that some signals are closer morphologically in relation to others. For example, ECG 8 is closer to ECG 15 than ECG 11, which represents the higher error value.

Not only the mean error (Fig. 10e), but also the standard deviation (Fig. 10f) pose low values for prediction, implying the characteristic nature of these signals, significantly different from individual to individual. These networks because specific for the ECG signal trait as they have a high error in all the other signals, both of the same and other types of biosignal.

## Conclusion and future remarks

With the achievements of this work we were able to replicate the morphology of the three presented biosignals using DNN architectures. The two main aspects of this architecture that differ from the bibliography, is the capacity to learn several and replicate several signals and that it is is blind, such as no features are given *a priori* about the input signal. The models also need just a few seconds of signal for training, taking into account that the ECG and EMG results only needed approximately 175 seconds and the RESP 350 s. The low error rate of the RESP and ECG also reflect the possibility of using this model to identify the source of these signals.

Some limitations also reside in the proposed architecture, such as: the sensitivity of the pre-processing to the noise disruptions with high amplitudes, as the whole signal may be compromised with the changes of the maximum and/or minimum values; the signal dimension reduction represents a loss of information; the fact that this architecture, in its core, is a state machine, and therefore possesses a limited number of states and, consequently, the memory capacity of coding an extensive signal; and, the computational burden, known in the RNN architectures, requiring a significant time to train data.

Further observation on the learned models will be directed to the search of the internal neural structures that generate the morphological aspects of the signal.

Future work will be in the creation of novel biometrics methods by selecting the model with lower loss for an input signal. The results displayed by the loss function could both evaluate the type of the signal or the source that originated it to identify the respective person.

Other direction will be in the detection of noisy areas of the signals, and if above a certain threshold, the learned model could be synthesized to replace the damaged time interval, increasing the capacity of feature extraction.

Other possible contributions of this paper is the application of this algorithm using TWs of normal against pathological physiological signals, as the deviation from the trained model could give a report regarding what segment the pathological events occur.

While exploring the inner workings of how the DNN model learns and generates the biosignals’ morphological characteristics it may be possible to generate valuable information on how to deliver novel procedures for decision making for support to the medical field.

## Footnotes

1 The used library to process the ANN algorithm was *Theano* [42], a Python library that allows to define, optimize and evaluate mathematical expressions involving multi-dimensional arrays efficiently. It has been one of the most used CPU and GPU mathematical compilers, and has been used to produce many state-of-the-art machine learning models since 1998 [42].
